# An attempt to improve recognition of fetal acidemia by a remodeled intrapartum cardiotocography classification: A case–control study

**DOI:** 10.1111/aogs.70287

**Published:** 2026-06-10

**Authors:** Maria Fogelberg, Johanna Wagner Bjurström, Charlotte Dahlbäck, Frida Ekengård, Gisela Rickle, Karin Källén, Andreas Herbst

**Affiliations:** ^1^ Department of Obstetrics and Gynecology Skane University Hospital Malmö Sweden; ^2^ Institution for Clinical Sciences Lund Lund University Lund Sweden; ^3^ Department of Obstetrics and Gynecology Holbæk Hospital, Region Zealand Holbæk Denmark; ^4^ Institution for Clinical Sciences Malmö Lund University Lund Sweden

**Keywords:** acidemia, Cardiotocography, electronic fetal monitoring, fetal heart rate, interpretation template

## Abstract

**Introduction:**

Current international (FIGO) and Swedish cardiotocography (CTG) interpretation templates exhibit limited diagnostic accuracy for the detection of fetal acidemia. The objective of this study was to develop a novel intrapartum CTG classification template incorporating CTG parameters demonstrated to be strongly associated with acidemia, and to evaluate the diagnostic performance of the models by assessing their sensitivity and specificity in identifying CTG recordings from neonates with acidemia.

**Material and Methods:**

A retrospective case–control study based on 1092 births in Region Skåne. The study material included 364 cases with umbilical cord blood pH <7.05, or <7.10 after 1st stage cesarean, and 728 controls with pH ≥7.15 and Apgar scores ≥9 at 5 and 10 min. CTG patterns were systematically evaluated across a set of predefined variables, and associations between specific patterns and fetal acidemia were quantified using ORs. Two models defining pathological patterns were created: Model 1 based on the presence of one of several listed criteria resulting in a positive test and Model 2 also including the presence of ≥2 B‐criteria resulting in a positive test. The main outcomes, sensitivity, and specificity to identify fetal acidemia at birth were calculated and compared with the current FIGO, NICE, and SWE guidelines.

**Results:**

The sensitivity to identify fetal acidemia was 86.3%, 86.5%, 50.8%, 57.1%, and 87.4% for Model 1, Model 2, FIGO, SWE, and NICE, respectively. The corresponding specificity was 77.9%, 76.4%, 92.6%, 91.2%, and 70.6%. The sensitivity to identify severe acidemia (pH <7.0) was 90.4% for Model 1 and for NICE; 64.4% for SWE, and 56.7% for FIGO. A variable not included in current classification systems, “fetal heart rate below baseline for >50% of the time during 30 minutes” was the criterion contributing most to the sensitivity.

**Conclusions:**

Our models show higher sensitivity but lower specificity for the defined outcome compared with SWE and FIGO, and similar sensitivity but higher specificity compared with NICE. Validating the model on a new material is essential before considering clinical use.

## Graphical abstract

Current CTG interpretation templates have shown low sensitivity or low specificity identifying fetuses with acidemia. This new interpretation template combined a high sensitivity with a high specificity. Validation on a new material is essential before considering clinical use.

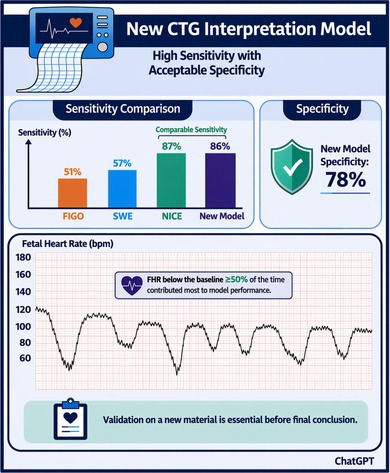

**Abbreviations**
Bpm: beats per minute
CI: confidence interval
CTG: cardiotocography
FHR: fetal heart rate
FIGO: The International Federation of Gynecology and Obstetrics
NICE: National Institute for Health and Care Excellence
OR: odds ratio
SWE: Swedish interpretation template from 2017

**Key message**

Our CTG interpretation templates show a good balance between sensitivity and specificity in identifying fetal acidemia, and incorporate a useful new parameter. Prospective validation is needed.

## 1 INTRODUCTION

Intrapartum cardiotocography (CTG) monitoring is intended to identify signs of fetal hypoxia, enabling timely interventions to prevent perinatal asphyxia.

Standardized interpretation templates have been developed to support the assessment of CTG tracings. The current Swedish guidelines (SWE) are based on the 2015 consensus recommendations from the International Federation of Gynecology and Obstetrics (FIGO).^1^ In 2022, the National Institute for Health and Care Excellence (NICE) updated its guidelines for CTG interpretation.^2^ All three templates employ a three‐tier classification system, categorizing CTG patterns as normal, suspicious, or pathological.

Several CTG interpretation templates currently in clinical use have demonstrated limited sensitivity for identifying fetuses with acidemia.^3^, ^4^, ^5^ By contrast, NICE guidelines have demonstrated high sensitivity (79%–98%) for detecting neonates with acidemia, albeit with relatively low specificity (59%–67%).^6^

Efforts to apply machine learning for the interpretation of fetal heart rate (FHR) patterns are promising, but to date, they have not consistently demonstrated superiority over conventional visual analysis. A notable study yielded a sensitivity of 79% and a specificity of 76% for identifying neonates with cord artery pH <7.05.^7^

Given that visual CTG assessment remains the clinical standard, and that existing interpretation templates do not simultaneously achieve both high sensitivity and high specificity, the present study aimed to develop an improved CTG interpretation template.

The objective was to develop a new intrapartum CTG interpretation template, based on criteria with sufficiently robust and clinically meaningful associations with fetal acidemia to warrant intervention. This set of criteria would constitute the category referred to as ‘pathological’ or ‘abnormal’ in a two‐tier or three‐tier classification system. The study aimed to evaluate the sensitivity and specificity of the complete model in identifying CTG tracings of neonates with acidemia.

A secondary objective was to compare the diagnostic performance of the newly developed model with three established classification systems: International Federation of Gynecology and Obstetrics (FIGO), the Swedish guidelines (SWE), and National Institute for Health and Care Excellence (NICE).

## 2 MATERIAL AND METHODS

This study included 1092 births from three labor units in Region Skåne, Sweden, between 2012 and 2017, comprising 364 cases with acidemia at birth and 728 controls. The dataset has previously been utilized to investigate associations between specific CTG patterns and neonatal acidemia at birth.^8^, ^9^ All deliveries at these labor units during the study period were eligible for inclusion in the study.

Inclusion criteria were singleton pregnancies in spontaneous or induced labor from gestational week 34 + 0. Cases were defined as neonates with umbilical cord blood pH <7.10 following cesarean delivery in the first stage of labor, or pH <7.05 after vaginal birth or cesarean delivery in the second stage. These thresholds were chosen as the aim of the model is to identify fetuses exposed to intrauterine hypoxia prior to the development of severe metabolic acidosis. Cases were included even when only a single umbilical cord blood pH value below the defined cut‐off was available. In such cases, it is not possible to ascertain whether the sample is arterial or venous. However, as umbilical arterial pH is physiologically lower than venous pH, a value below the defined cut‐off would imply that the arterial value is below this cut‐off regardless of if the sample is arterial or venous. Thus, a value below the cut‐off will meet the inclusion criterion if it is arterial, and if it is venous, the corresponding arterial pH would be expected to be even lower and thus also meet the inclusion criteria. The different cut‐offs for first‐stage cesarean deliveries and second‐stage births were motivated by the physiological decline in pH during the second stage of labor.^10^ A pH <7.10 in the first stage was considered indicative of reduced fetal reserve and a higher likelihood of progression to more severe acidemia if labor had continued, whereas a pH of 7.05–7.09 after vaginal birth was considered more likely to reflect a shorter duration of hypoxic exposure. For both pH thresholds, a significantly increased risk of severe neonatal outcomes has been reported, although with low absolute incidences.^11^ These cut‐offs were considered to represent the lowest pH levels at which a warning signal would still allow timely clinical intervention.

Controls were defined as neonates with umbilical artery and vein pH values ≥7.15 and Apgar score ≥9 at five and 10 min. A minimum arterial–venous difference of 0.02 was required to ensure accurate vessel identification and avoid misclassification. For each case, the first two eligible neonates born subsequently at the same hospital served as controls. Background characteristics are summarized in Table 1. No matching or adjustment for key obstetric factors was performed, as the primary objective was to assess the association between CTG patterns and neonatal acidemia, irrespective of underlying obstetric conditions.

**TABLE 1:** Summary of background data of cases and controls.

	Cases *n* (%)	Controls *n* (%)	Missing data *n*	Total *n*
Total	364	728		1092
Primipara	225 (61.0)	356 (48.6)	0	1092
Instrumental delivery	88 (24.2)	45 (6.2)	0	1092
Cesarean section	94 (25.8)	12 (1.6)	0	1092
Breech	2 (0.5)	1 (0.1)	0	1092
Post‐term birth ≥42 + 0	28 (7.7)	50 (6.9)	0	1092
Preterm birth <37 + 0	8 (2.2)	18 (2.5)	0	1092
Birthweight >4.5 kg	13 (3.6)	18 (2.5)	3	1089
Birthweight <2.5 kg	5 (1.4)	10 (1.4)	3	1089
Epidural	159 (43.7)	215 (29.5)	0	1092
Oxytocin augmentation	202 (55.5)	252 (34.6)	0	1092
Meconium‐stained amniotic fluid	96 (26.4)	137 (18.8)	4	1088
Fever/Infection	11 (3.0)	8 (1.1)	0	1092
Preeclampsia	15 (4.1)	16 (2.2)	0	1092
Diabetes	22 (6.0)	19 (2.6)	0	1092
Smoking	20 (5.5)	54 (7.4)	18	1075
BMI <25	209 (57.4)	433 (59.5)	29	1063
BMI >30	47 (12.9)	80 (11.0)	29	1063
5‐min Apgar score <7	47 (12.9)	0	1	1091
Cord artery pH available	344 (94.5)	728 (100)	20	1072
Cord vein pH available	344 (94.5)	728 (100)	20	1072
Both artery +vein pH available	304 (83.5)	728 (100)	40	1052
Cord artery or vein pH <7.00	104 (28.6)	0	0	1092
Cord artery or vein pH <7.05	328 (90.1)	0	0	1092
Cord artery or vein base deficit >12 mmol/L	96 (26.7)	2 (0.3)	13	1079

### 2.1 Assessment of association between discrete CTG patterns and acidemia

The CTG recordings of the last hour before birth were analyzed, except for controls matched to first stage cesarean cases, where traces from the same cervical dilation as the corresponding cases were analyzed. All assessments were performed independently by two of the authors, blinded to group allocation and clinical information other than stage of labor. Disagreements in interpretation of any CTG variable were resolved by a third, and if necessary, a fourth assessor, according to majority rule.

Variables evaluated included baseline FHR, variability, decelerations, and contraction patterns. Odds ratios (ORs) for the association between individual variables, or combinations thereof, and acidemia were calculated with 95% confidence intervals (CIs).

Baseline FHR: All baseline levels persisting for ≥10 min were recorded. For the primary univariable analyses, the reference group was defined as baseline of 110–160 bpm, consistent with the definition of a normal baseline in current classifications.^12^

Variability: Normal variability was defined as 5–25 bpm. Reduced variability (2–4 bpm) was considered when lasting ≥50 min (according to FIGO and NICE) and only when not accompanied by sporadic accelerations as recent evidence demonstrates that this rare combination is not associated with acidemia.^8^ Previous studies have shown that accelerations in the last hour of labor are negatively associated with acidemia^13^ and that their absence may indicate increased fetal risk.^14^ However, the interval since the last acceleration has not been shown to correlate with scalp blood lactate concentrations.^15^

Increased variability was defined using a 10‐min time threshold, following NICE recommendations^16^ and since previous studies have identified increased variability as an early marker of fetal hypoxia.^17^, ^18^

Absent variability (<2 bpm) is specified as a pathological criterion in SWE guidelines, though no time limit is defined. In the guidelines for STAN monitoring (2007), absent variability (with or without decelerations or bradycardia) has been defined as a “preterminal” pattern, a fourth tier more severe than “pathological”.^19^, ^20^ Absent variability for 10 min has shown a strong association with severe metabolic acidemia in prior work^21^ and is generally observed as a late sign in the hypoxic process. In that study, the mean duration of absent variability in cases with cord artery pH <7.05 was 5 min (unpublished data). A 5‐min minimum duration for absent variability was therefore pragmatically applied in the present study.

Contraction patterns: An elevated contraction rate was defined as >5 contractions per 10 min on average during 30‐min and/or ≥6 contractions per 10 min for 20 consecutive minutes.

Decelerations: Associations between different types and characteristics of decelerations and acidemia were previously examined using the same dataset and methodology.^9^ The findings from that study were used to define the deceleration patterns incorporated into the new models.

### 2.2 Creation of prediction models

Two novel CTG interpretation models were developed, each defining criteria for pathological (abnormal) patterns.

- Model 1: Pathological if any single A‐criterion is present.
- Model 2: Pathological if either one A‐criterion or two B‐criteria are present.

CTG patterns associated with acidemia with OR about 5 or more were considered for inclusion as A‐criteria; patterns with OR about 3 or more were considered as B‐criteria in Model 2. Rare, established pathological patterns with insufficient incidence to evaluate statistically were also included to ensure clinically relevant criteria were not excluded. Based on our previous work and the criteria described above, decelerative patterns listed in Table 2 were included as determinators of pathological classification. Criteria for other CTG variables were defined based on the present results (see Section 3).

**TABLE 2:** Association between different CTG patterns and acidemia at birth, and prevalences of these patterns in neonates with acidemia and controls.

	OR	*p*‐value	Prevalence (%) neonates without acidemia, *N* = 728	Prevalence (%) Neonates with acidemia, *N* = 364
Baseline: 110–160 bpm	*Reference*		92.6	65.1
Baseline >170 bpm	8.8 (4.4–18)	<0.0001	1.5	9.3
Baseline 161–170 bpm	3.8 (2.3–6.2)	<0.0001	4.1	11.0
Baseline 100–109 bpm	5.0 (2.1–12)	0.0004	1.1	3.8
Baseline <100 bpm	23 (8.9–58)	<0.0001	0.7	11.0
Baseline increase ≥20 bpm *Reference: Absence of this criterion*	4.8 (3.1–7.5)	<0.0001	4.5	18.7
Variability: 5–25 bpm	*Reference*		97.4	81.6
Variability <5 bpm without accelerations ≥50 min	3.6 (1.6–8.1)	0.002	1.4	4.1
Variability <2 bpm ≥5 min	30 (11–85)	<0.0001	0.5	13.7
Variability >25 bpm ≥10 min	3.3 (1.05–11)	0.04	0.7	1.9
Decelerations: *Reference for each criterion is the absence of the same criterion*				
Repetitive late decelerations >20 min (1)	19 (9.7–37)	<0.0001	1.4	20.9
≥5 late decelerations during 1 h (1)	9.0 (6.1–13)	<0.0001	5.8	35.4
Repetitive combined decelerations >20 min (1)	6.4 (3.1–13)	<0.0001	1.4	8.2
≥5 combined decelerations during 1 h (1)	4.2 (2.7–6.4)	<0.0001	4.9	17.9
≥3 variable dec >60 s with variability <2 bpm within the decelerations	17.4 (6.1–50)	<0.0001	0.5	8.8
Repetitive variable decelerations >60 s with baseline >160 or variability <5 between dec >20 min (1)	3.3 (1.5–7.5)	0.003	1.4	4.4
Repetitive variable decelerations >60 s > 30 min (1)	1.7 (1.0–3.0)	0.07	3.8	6.3
≥10 variable decelerations >60 s during 1 h (1)	2.7 (1.5–4.7)	0.0003	3.4	7.7
Prolonged deceleration ≥5 min (before the last 10 min) (1)	12 (7.9–19)	<0.0001	3.7	31.6
≥3 prolonged decelerations 3–5 min (1)	10 (4.3–25)	<0.0001	0.8	8.0
1 prolonged deceleration 3–5 min (before the last 10 min) (1)	4.9 (3.6–6.7)	<0.0001	11.1	38.2
≥50% below baseline during 30 min due to decelerations (1)	14 (10–19)	<0.0001	10.7	62.4
Contractions: *Reference is the absence of the criterion below*				
>5 contractions/10 min during >30 min OR ≥ 12 contractions during 20 min.	2.9 (2.1–3.9)	<0.0001	13.5	30.8

*Note*: (1) Fogelberg M, Dahlbäck C, Ekengård F, Rickle G, Herbst A. Association between different types and characteristics of fetal deceleration during labour and neonatal acidemia at delivery: A case–control study. *Eur J Obstet Gynecol Reprod Biol X*. 2025 Apr 25;26:100389. https://doi.org/10.1016/j.eurox.2025.100389. PMID: 40385099; PMCID: PMC12083902.

### 2.3 Assessment of sensitivity and specificity

The diagnostic performance of the new models was analyzed and compared with three established CTG classification systems—FIGO, NICE, and SWE^1^, ^2^, ^22^ by comparing the sensitivity and specificity for detecting neonates with acidemia using the classification pathological. In addition, the sensitivity for detecting severe acidemia at birth (pH <7.0) was also evaluated.

### 2.4 Statistical analyses

Univariate odds ratios with 95% CI, sensitivity and specificity with 95% CI, and chi‐square *p*‐values were calculated using MedCalc for Windows, version 23.3.7 (MedCalc Software, Ostend, Belgium). Given the sample size, multiple logistic regression was not performed to optimize model selection. Analyses were therefore limited to the two primary models and a small number of secondary tests deemed justified. The contribution of “new” parameters was assessed by evaluating changes in model performance when these variables were omitted. In addition, we performed exploratory analyses using univariable and multivariable logistic regression models to gain further insight into which variables contributed most to the discriminatory performance. These analyses were conducted while acknowledging the limitations related to the sample size and the potential risk of overfitting. In this analysis, each variable was treated as binary, that is, the reference for each variable was the absence of the same variable. Forward selection was used, based on likelihood ratio (LR) statistics from univariable models. In intermediate models, variables were excluded if *p* > 0.20 or if inclusion of the variable did not improve model fit as assessed by the LR test. The coefficients from the final multivariable model were used to calculate predicted odds for each individual. These estimates were then used to construct a receiver operating characteristic (ROC) curve. The area under the curve (AUC) and its 95% CI were estimated using the method of DeLong et al.^23^

A post hoc analysis was conducted to assess the impact of the variable “≥50% below baseline for ≥30 min” across the FIGO, NICE, and SWE templates. Sensitivity and specificity were recalculated for all systems after incorporating this variable as a criterion for pathological CTG classification.

Additional analyses were also performed to evaluate interobserver agreement for key CTG variables classified as pathological in Model 1 and on the overall classification of CTG as pathological according to the new model, using Cohens kappa.

## 3 RESULTS

### 3.1 Association between discrete CTG patterns and acidemia

Table 2 presents ORs (95% CI) for the association between individual CTG variables and acidemia. Previously reported data on decelerations^9^ are included for reference.

Baseline FHR: A baseline fetal heart rate >170 bpm was strongly associated with acidemia (OR 8.8; 95% CI: 4.4–18) and occurred in 1.5% of controls. A baseline of 161–170 bpm showed a moderate association (OR 3.8; 95% CI: 2.3–6.2), and the prevalence in controls was 4.1%. A baseline of <100 bpm was also strongly associated with acidemia (OR 23; 95% CI: 8.9–58). Baseline values of 100–109 bpm demonstrated a moderate association (OR 5.0; 95% CI: 2.1–12). A baseline increase ≥20 bpm (NICE “amber” criterion) had an OR of 4.8 (3.1–7.5) and occurred in 4.5% of controls. After excluding traces with baseline >160 bpm, the OR declined to 2.8 (1.6–4.7).

Variability: Reduced variability (<5 bpm) without accelerations for >50 min was associated with acidemia (OR 3.6; 95% CI: 1.6–8.1) and was present in 1.4% of controls. Reduced variability lasting 30–50 min (NICE amber criterion) was not associated with acidemia (OR 1.1; 95% CI: 0.55–2.1).

Absent variability (<2 bpm) for ≥5 min was strongly associated with acidemia (OR 30; 95% CI: 11–85) and had low prevalence among controls (0.5%). Increased variability (>25 bpm) demonstrated a modest association (OR 3.3;95% CI: 1.05–11) and was rare (0.7%) among controls. Sinusoidal variability was only observed in one case.^24^

Decelerations and contractions: Patterns were defined based on previous analyses.^9^

### 3.2 Proposed CTG interpretation models

Model 1 classifies a CTG as pathological if any single criterion is present. Pathological (A) criteria included: baseline >170 bpm or FHR <100 bpm; variability <5 bpm with <2 accelerations for >50 min; variability >25 bpm for ≥10 min; absent variability (<2 bpm) for ≥5 min; sinusoidal pattern; any types of decelerations specified in Table 3; FHR below the baseline ≥50% of the time for ≥30 min.

**TABLE 3:** Prediction models. Model 1 in which the presence of one criterion resulted in a positive test (pathological pattern) and Model 2 in which also the presence of two B‐criteria resulted in a positive test.

Model 1	Pathological if any of:
Baseline	Baseline >170 bpm^a^ FHR <100 bpm
Variability	Variability <5 bpm with <2 accelerations ≥50 min Variability <2 bpm ≥5 min Variability >25 bpm ≥10 min Sinusoidal pattern
Decelerations	Repetitive late dec >20 min OR ≥5 late dec during 1 h Repetitive combined dec >20 min OR ≥5 combined dec during 1 h ≥3 variable dec >60 s with variability <2 bpm within the dec during 1 h Repetitive variable dec >60 s with FHR >160 bpm or variability <5 bpm between the dec >20 min One prolonged dec >5 min ≥3 prolonged dec 3–5 min during 1 h FHR below the baseline ≥50% of the time for ≥30 min

Model 2	Pathological if any of: A‐criteria^a^	Pathological if two of: B‐criteria
Baseline	Baseline >170 bpm FHR <100 bpm	Baseline 160–169 bpm or ≥ 20 bpm increase in baseline or baseline 100–109 bpm
Variability	Variability <5 bpm with <2 accelerations ≥50 min^b^ Variability <2 bpm ≥5 min Variability >25 bpm ≥10 min Sinusoidal pattern	
Decelerations	Repetitive late dec >20 min OR ≥5 late dec during 1 h Repetitive combined dec >20 min ≥3 variable dec >60 s with variability <2 bpm within the dec during 1 h Repetitive variable dec >60 s with FHR > 160 bpm or variability <5 bpm between dec >20 min One prolonged dec >5 min ≥3 prolonged dec 3–5 min during 1 h FHR below the baseline ≥50% of the time for ≥30 min	≥5 combined dec during 1 h^c^ Repetitive variable dec >60 s > 30 min OR ≥10 variable dec >60 s during 1 h One prolonged dec >3 min
Contractions		≥12 contractions >20 min or >5 contractions in 10 min >30 min

^a^ As an alternative baseline >160 bpm was tested.

^b^ As an alternative variability <5 bpm without accelerations ≥50 min was tested as a B‐criterion.

^c^ As an alternative ≥5 combined decelerations during 1 h was tested as an A‐criterion.

Model 2 classifies a CTG as pathological if either one A‐criterion or two B‐criteria are present. B‐criteria included: baseline 160–169 bpm or 100–109 bpm or ≥20 bpm increase in baseline; ≥5 combined decelerations per hour (in this model excluded from the A‐criteria); repetitive variable decelerations >60 s for >30 min or ≥ 10 per hour; one prolonged deceleration >3 min; uterine contraction rate above specified thresholds. The full models are summarized in Table 3.

### 3.3 Diagnostic performance

The sensitivity and specificity of the new models and the existing templates are presented in Table 4. Both new models had a sensitivity of 86%. The specificity was 78% for Model 1 and 76% for Model 2. Compared with established templates, sensitivity for detecting acidemia at birth was higher for both models compared with FIGO and SWE and was comparable to NICE. The specificity for both models was significantly lower than for FIGO and SWE and higher than that of NICE.

**TABLE 4:** Sensitivity and specificity (with 95% confidence intervals) to identify neonates with acidemia by the CTG classification ´pathological´ for Models 1 and 2, FIGO 2015, NICE 2022 and the current Swedish classification system (SWE 2017).

	True‐positive (*N*)	False‐positive (*N*)	True‐negative (*N*)	False‐negative (*N*)	Sensitivity (95% CI)	*p*‐value (compared with Model 1)	Specificity (95% CI)	*p*‐value (compared with Model 1)
Model 1	314	161	567	50	86.3 (82.3–89.6)		77.9 (74.7–80.9)	
Model 2	315	172	556	49	86.5 (82.6–89.9)	0.91	76.4 (73.1–79.4)	0.49
FIGO 2015	185	54	674	179	50.8 (45.6–56.1)	<0.0001	92.6 (90.4–94.4)	<0.0001
NICE 2022	318	214	514	46	87.4 (83.5–90.6)	0.59	70.6 (67.1–73.9)	0.0015
SWE 2017	208	64	664	156	57.1 (51.9–64.3)	<0.0001	91.2 (88.9–93.2)	<0.0001

To evaluate the contribution of “variability <2 bpm during ≥5 min,” the model was tested without this variable, which did not alter sensitivity or specificity significantly.

For the same reason, the model was tested without the variable “FHR below the baseline for 50% of the time during 30 min.” Removal of this criterion resulted in a significantly decreased sensitivity to 79.4% (74.9–83.4), and a significantly increased specificity (81.9%; 78.9–84.6).

**FIGURE 1:**
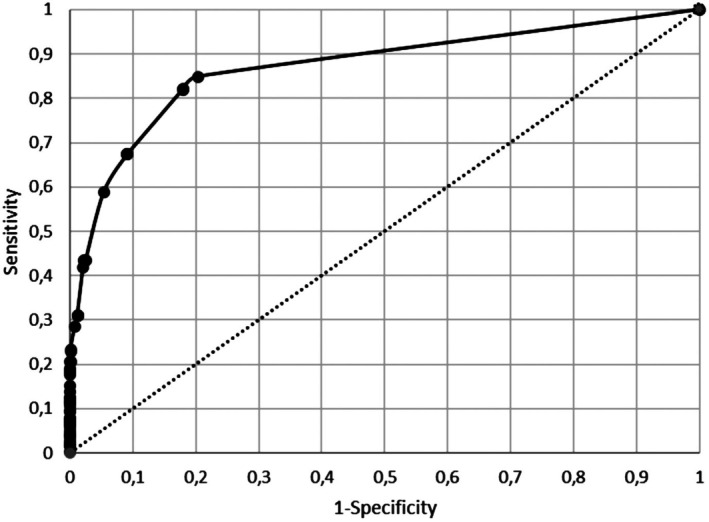
Receiver operating characteristic (ROC) curve based on the coefficients from the final multivariable model given in Table 5. The area under the curve (AUC) and its 95% confidence interval were estimated using the method of DeLong et al. (a). AUC = 0.87 (0.84–0.89). (a) DeLong ER, DeLong DM, Clarke‐Pearson DL. Comparing the areas under two or more correlated receiver operating characteristic curves: A nonparametric approach. *Biometrics*. 1988;44(3):837–45.

Logistic regression analysis identified baseline >170 bpm, variability <2 bpm for ≥5 min, and ≥3 variable decelerations >60 s with variability <2 bpm within the deceleration as having the highest adjusted associations to acidemia (ORs). The variable contributing most to model performance was “FHR below the baseline ≥50% of the time for ≥30 min.” Four variables did not contribute independently to the model: variability <5 bpm for >50 min; variability >25 bpm for ≥10 min; sinusoidal pattern; and repetitive variable decelerations >60 s with FHR >160 bpm or variability <5 bpm between the decelerations. All results are presented in Table 5. ROC analysis showed an AUC of 0.87 (95% CI: 0.84–0.89; Figure 1).

**TABLE 5:** Odds ratios comparing cases and controls for each criterion in Model 1, derived from univariable and multivariable logistic regression models.

	Cases (*N* = 364)	Controls (*N* = 728)	Univariable models			Final, multivariable^b^ model	
	*n* (%)	*n* (%)	OR	95% CI	LR^a^ chi‐square	OR	95% CI
Baseline >170 bpm	34 (9.3)	11 (1.5)	6.7	3.4–13.4	35.1	11.6	5.2–26.2
Baseline <100 bpm	40 (11.0)	5 (0.7)	17.9	7.0–45.6	63.2	4.3	1.3–14.2
Variability <5 bpm without accelerations	15 (4.1)	10 (1.4)	3.1	1.4–6.9	7.6	—	—
Variability <2 bpm ≥5 min	50 (13.7)	4 (0.5)	28.8	20.3–80.5	89.1	11.3	4.5–28.7
Variability >25 bpm ≥10 min	7 (1.9)	5 (0.7)	2.8	0.9–9.0	3.2	—	—
Sinusoidal pattern	1 (0.3)	0 (0.0)	—	—	2.2	—	—
≥5 late dec during 1 h	129 (35.4)	42 (5.8)	9.0	6.1–13.1	153.4	4.7	2.9–7.7
≥5 combined dec during 1 h	65 (17.9)	37 (5.1)	4.1	2.7–6.2	43.6	2.5	2.3–4.7
Repetitive variable dec >60 s with FHR >160 bpm or variability <5 bpm between the dec >20 min	16 (4.4)	10 (1.4)	3.3	1.5–7.4	8.8	—	—
≥3 variable dec >60 s with variability <2 bpm within the decelerations	32 (8.8)	4 (0.5)	17.4	6.1–49.7	50.2	11.3	3.6–35.5
≥3 prolonged decelerations 3–5 min	29 (8.0)	6 (0.8)	10.4	4.3–25.3	37.8	5.2	2.0–13.3
Prolonged dec ≥5 min (before the last 10 min)	116 (31.9)	27 (3.7)	12.1	7.8–18.9	161.3	4.5	2.4–8.6
≥50% below baseline during 30 min	227 (62.4)	78 (10.7)	13.8	10.1–18.9	315.7	4.8	3.1–7.3

^a^ Likelihood ratio Chi‐square for the univariable models.

^b^ The model included all variables listed in the column. Forward selection was based on likelihood ratio (LR) statistics from univariable models. In intermediate models, variables were excluded if *p* > 0.20 or if the inclusion of the variable did not improve model fit as assessed by the likelihood ratio test.

Post hoc analyses: For the detection of severe acidemia (pH <7.0), the sensitivity was 90.4% (83.0–95.3) for Model 1 and for NICE, compared with 64.4% (54.4–73.6) for SWE and 56.7% (46.7–66.4) for FIGO.

The impact of the criterion “FHR below baseline ≥50% for ≥30 min” was evaluated across FIGO, SWE, and NICE templates. Sensitivity and specificity were recalculated after incorporating this variable as a pathological criterion for each system. It demonstrated minimal impact on the diagnostic performance of NICE. Sensitivity increased and specificity decreased for FIGO and SWE (Table S1); however, both templates still demonstrated lower sensitivity and higher specificity than that of NICE and our proposed template.

When suspicious patterns were included as a positive test alongside patterns classified as pathological, the sensitivity increased for FIGO to 92.9% (89.7–95.3) for SWE to 73.4% (68.5–77.8) and for NICE to 96.1% (93.6–97.9). The corresponding figures for specificity were 36.3% (32.8–39.9) for FIGO, 73.4% (68.5–77.8) for SWE and 37.5% (34.0–41.1) for NICE.

Interobserver agreement: Overall, interobserver agreement between the two primary assessors was moderate to substantial for the discrete CTG patterns evaluated except for increased variability (>25 bpm) and for the combination of variable decelerations >60 s and a baseline >160 bpm or variability <5 bpm, for which agreement was fair (Table S2). For overall CTG classification, interobserver agreement was substantial, with 87.3% agreement and a kappa coefficient of 0.74.

## 4 DISCUSSION

This study evaluated two novel intrapartum CTG classification models that identified neonates with acidemia with a sensitivity comparable to that of NICE guidelines and with higher specificity. Both models, as well as NICE, demonstrated higher sensitivity and lower specificity than FIGO and the Swedish guidelines.

Of the proposed models, Model 1 is considered preferable due to its simplicity, as it requires only a single criterion for classification as pathological, which may enhance usability and consistency in clinical interpretation.

Importantly, the difference in sensitivity persisted even for severe acidemia, with both the proposed Model 1 and NICE demonstrating higher sensitivity than FIGO and SWE. Expanding the definition of a positive test to include “suspicious” patterns markedly reduced specificity for FIGO and NICE, rendering these templates clinically impractical, as only 36%–37% of CTG traces were then classified as normal. For SWE, specificity was higher (81.5%), but sensitivity was still lower (73.4%) than for a pathological pattern according to Model 1 and NICE.

Previous studies have reported variable diagnostic performance of intrapartum CTG classification systems. Zamora Del Pozo et al. reported sensitivities for identifying neonates with cord artery pH <7.10 of 24% for FIGO, 39% for NICE (2017), 79% for Chandraharan guidelines, and 15% for ACOG category III.^5^ Marti Gamboa et al. reported sensitivities of 43.6% for FIGO and 36.3% for the Parer and Ikeda five‐tier system, with corresponding specificities of 82.5% and 88.0%.^4^ In our study, the sensitivity and specificity of FIGO were consistent with previous findings reported by Kling et al.^6^ The sensitivity of NICE (2022) was comparable to the smaller study by Kling et al., while specificity was higher in the present study.

We consider high sensitivity essential for a CTG interpretation template intended to support safe intrapartum care. While specificity is also important, particularly to avoid unnecessary interventions, abnormal CTG findings do not necessarily always need to prompt immediate or invasive interventions. According to the clinical situation, proximity to expected birth, and individual assessment of FHR pattern development, alternatives may include optimization of the situation, a second opinion by a more experienced colleague, or fetal scalp blood analysis.

Interobserver agreement for discrete CTG variables and overall classification in this study was comparable to that reported in previous evaluations of agreement when classifying CTG patterns according to FIGO, NICE, and SWE interpretation systems.^25^, ^26^, ^27^

The logistic regression analysis indicated that all but four CTG features accounted for the model's discriminatory performance. The variables, baseline >170 bpm, absent variability, and variable decelerations with absent variability within decelerations showed the highest independent association to acidemia. By contrast, “FHR below the baseline ≥50% of the time” contributed most to model performance, indicating that this is a frequently occurring deviation in fetuses exposed to hypoxia. Four variables did not contribute independently to the model, which may indicate that these are infrequent or have limited predictive value, or that acidemia was captured by other correlated features. The overall discriminatory ability was good (AUC 0.87) but requires validation in independent cohorts.

To our knowledge, this is the first attempt to develop an intrapartum CTG interpretation template based not only on the strength of association between individual CTG variables and acidemia but also on the diagnostic performance of the complete model prior to consideration of clinical implementation.

The relatively large number of cases and controls allowed estimation of sensitivity and specificity with reasonably narrow confidence intervals for most variables. However, the sample size was insufficient to reliably assess associations for rare CTG patterns, and some variables were therefore included despite modest observed associations with acidemia.

All CTG traces were independently assessed by at least two observers, which is likely to enhance external validity compared with single‐observer assessments. Only the final hour before birth was analyzed. Inclusion of earlier CTG recordings might have reduced specificity, a limitation that would then apply to all interpretation systems.

Another potential limitation is the possibility of confounding. Risk factors for fetal hypoxia, such as placental insufficiency and fetal growth restriction, may influence both the occurrence of CTG abnormalities and the risk of fetal acidemia. We do not consider such factors as potential confounders, but factors influencing the baseline risk of acidemia as well as CTG abnormalities. An increased baseline risk should not affect sensitivity or specificity, but influences positive and negative predictive values. Since the primary aim of this study was to evaluate the association between CTG patterns and acidemia regardless of underlying risk factors, no adjustments for these variables were performed, and as this is a case–control study, predictive values are not assessed. The duration of the second stage may be a more complex variable, possibly contributing to fetal acidemia through two mechanisms. It may increase the risk of fetal hypoxia and thereby for acidemia at birth, and may also contribute to fetal acidemia through maternal anaerobic metabolism.^28^, ^29^, ^30^, ^31^, ^32^ A prolonged second stage resulting in acidemia by fetal hypoxia may be considered as a mediator. By contrast, a prolonged second stage resulting in acidemia by maternal anaerobic metabolism may be considered as a potential confounder, *if* CTG patterns also are affected by maternal anaerobic metabolism. Yet, we do not consider that prolonged maternal pushing would be enough to result in a cord blood pH value below 7.05. A recent systematic review reported that none of eight studies found an association between duration of the second stage of labor and acidemia at birth,^33^ whereas a study from our institution found a modest association between pushing time and acidemia.^30^ Prolonged second stage of labor has also been associated with low Apgar scores and admission to neonatal intensive care.^33^

The present study was based on clinical data in which interventions were performed in response to suspected fetal distress. Although this reflects real‐world practice, it precludes assessment of the natural progression of abnormal CTG patterns. Intrapartum interventions in response to abnormal CTG likely altered progression toward acidemia, as fetuses with pathological CTG may have undergone expedited delivery before developing significant acidemia. This may have resulted in an apparent increase in false‐positive classifications and thus an underestimation of specificity. At the same time, interruption of this progression may also limit the ability to fully assess the sensitivity of CTG in identifying fetuses that would have developed acidemia if labor had continued. This reflects an inherent limitation of studies conducted in contemporary clinical settings, where withholding intervention would be unethical.

Since the study population partially overlaps with previously published datasets,^3^, ^34^ the present findings do not substantially strengthen the evidence base regarding existing templates such as FIGO and SWE. Furthermore, the models were developed and tested within the same dataset, introducing a risk of overfitting and limiting generalizability. This approach may be associated with somewhat optimistic estimates of diagnostic performance. Thus, results must be validated on a new material before firm conclusions about the diagnostic validity may be drawn.

Application of the proposed models requires specific training in the interpretation of the included CTG patterns. Diagnostic performance may vary with observer experience, and different validation studies are therefore warranted. Planned evaluations include both validation by using a new dataset and external validation by using different interpreters at centers in two countries. We welcome other researchers to evaluate the model on retrospective data from their clinical settings.

Subgroup analyses were not performed due to limited representation of certain clinical conditions, such as fetal growth restriction and chorioamnionitis, and since the model was designed for general use from 34 weeks of gestation. Future studies are planned to evaluate performance in earlier gestation. As with all CTG interpretation systems, clinical context and obstetric risk factors must be considered.

The new criterion “FHR below the baseline for 50% of the time during 30 min” contributed substantially to increased sensitivity but reduced specificity. This criterion is related to frequent and/or prolonged decelerations and reflects a high burden of time spent below baseline. Unlike morphology‐based definitions relying on individual decelerations, the new criterion is a quantitative, time‐based measure capturing the cumulative duration of FHR below baseline. This pattern was present in 62% of cases and demonstrated a higher sensitivity to identify acidemia than all FIGO pathological criteria combined (51%). It should, however, be noted that the majority of CTG traces in the current study were from the last hour before vaginal birth, and that this type of pattern is not a typical finding in case of fetal distress before the second stage of labor.

Absent variability ≥5 min showed a strong association with acidemia; still, its exclusion did not significantly affect model sensitivity. This likely reflects that this pattern represents a late manifestation of fetal hypoxia, typically preceded by other abnormalities already included in the model.

Increased variability was the variable associated with the lowest interobserver agreement in the present study. A possible explanation for this finding is the lack of a precise definition regarding how the duration of increased variability should be assessed; whether the duration should be defined as a period with variability continuously exceeding 25 bpm, or as a period during which variability repeatedly exceeds 25 bpm without clearly returning to normal in between. In future studies, a more explicit definition of this parameter may be necessary.

Some CTG abnormalities develop gradually over time, reflecting a progressive deterioration in fetal condition, whereas others may occur abruptly and may be transient in nature. The clinical implications of a pathological CTG classification may therefore vary depending on the temporal pattern of abnormalities. A pattern that develops slowly and persists may indicate an ongoing hypoxic process, while brief or transient abnormalities may be of limited clinical significance. CTG monitoring should therefore be viewed as a dynamic tool rather than a static classification system. Its primary role is to serve as an early warning signal of potential risk to the fetus, enabling clinical assessment and appropriate interventions. Such interventions may include corrective measures, such as maternal repositioning, reduction or discontinuation of uterotonic agents, or other supportive actions and not only immediate delivery. Importantly, if the CTG pattern normalizes following such measures, no further intervention is usually required beyond continued CTG surveillance. Ongoing reassessment of CTG findings and consideration of the clinical context are essential for decision‐making during labor.

CTG interpretation templates are intended as tools to support clinicians in identifying fetuses at risk. Preferably, CTG patterns should be evaluated in the clinical context for the individual patient including assessment of risk factors, development of patterns over time, physiological grounds for specific patterns, and expected remaining time to birth. However, especially for clinicians with limited experience, structured classification systems may be an important aid. In a previous study, we found that registrars rely largely on the current guideline when screening CTG patterns.^35^ In this context, a high sensitivity of the template may be a prerequisite for the primary recognition of cases at risk.

While most intrapartum CTG interpretation systems employ three tiers, the present study focused exclusively on identifying patterns associated with the highest risk. The role of an intermediate category for enhanced surveillance was not addressed and warrants further investigation.

If validated in independent datasets, the proposed model might be considered for clinical implementation after a structured training program and feasibly by a cluster‐randomized trial, evaluating the impact on clinically relevant outcomes.

## 5 CONCLUSION

Both proposed interpretation models demonstrated high sensitivity with fair specificity in this dataset. Model 1 may be advantageous due to its simplicity. Compared with FIGO and SWE, the new model showed higher sensitivity and lower specificity while maintaining sensitivity comparable to NICE with improved specificity.

These findings should be interpreted with caution, as they are based on a single dataset and a limited number of observers and should therefore be considered hypothesis‐generating. Further validation, using new CTG datasets and involving observers with varying levels of clinical experience, is required prior to making a conclusion about the validity and clinical usability of the proposed model.

## AUTHOR CONTRIBUTIONS

Conceptualization, investigation, project administration: AH, MF. Funding acquisition: AH, FE. CTG assessment: MF, JB, FE, CD, GR, and AH. Methodology and analysis: MF, KK, and AH. Writing original draft: MF. Manuscript review and editing: All authors.

## FUNDING INFORMATION

The study was supported by research grants from Region Skåne and LÖF, the Swedish patient insurance company.

## CONFLICT OF INTEREST STATEMENT

The authors have no financial interests or personal relationships influencing the work reported in this article.

## ETHICAL APPROVAL

This study was conducted in accordance with the principles of the Declaration of Helsinki and ethical approval was obtained from the Regional Ethical Review Board in Lund, Dnr 2016/371, May 24, 2016. The requirement for written informed consent was waived, as all data were analyzed in a manner that preserved participant privacy.

## Supporting information


**Table S1.** Sensitivity and specificity of three current CTG interpretation guidelines to identify neonates with acidemia, after the investigational addition of the criterion “fetal heart rate >50% below the baseline during 30 minutes” to the respective guideline. For the proposed Model 1, this criterion is included as one of the features defining a “pathological” pattern.

**Table S2.** Agreement between two assessors in interpretation of different CTG patterns included as “pathological” in Model 1, and agreement in classification pathological according to the new model. All 1092 traces are included except for the variable “Variability <5 bpm without accelerations ≥50 min” for which only registrations of 60 min were included.

## Data Availability

Our ethical approval regrettably does not include sharing data on request.
