# Supplementary material for: An attempt to improve recognition of fetal acidemia by a remodeled intrapartum cardiotocography classification: A case–control study
Source: Acta Obstet Gynecol Scand. 2026 Jun 10;105(9):1749–60. doi: 10.1111/aogs.70287 (PMC13394735; doi:10.1111/aogs.70287)
Supplement: Supplementary file 1 [file AOGS-105-1749-s001.docx]

| Table S2 Agreement between two assessors in interpretation of different CTG patterns included as ´pathological´ in Model 1, and agreement in classification pathological according to the new model. All 1092 traces are included except for the variable “Variability <5 bpm without accelerations ≥50 min” for which only registrations of 60 minutes were included. | | |
| --- | --- | --- |
|  | **Agreement  (%)** | **Kappa index**  **(95% CI)** |
| Baseline >170 bpm | 98.0% | 0.74 (0.63-0.84) |
| Baseline <100 bpm | 97.7% | 0.74 (0.64-0.84) |
| Variability <5 bpm without accelerations ≥50 min | 96.9% | 0.59 (0.45-0.73) |
| Variability <2 bpm ≥5 min | 97.2% | 0.76 (0.68-0.74) |
| Variability >25 bpm ≥10 min | 93.6% | 0.24 (0.13-0.35) |
| ≥5 late decelerations during 1 h | 90.9% | 0.66 (0.60-0.72) |
| ≥5 combined decelerations during 1 h | 92.6% | 0.53 (0.43-0.62) |
| ≥3 variable dec >60 s with variability <2 bpm within the decelerations (1) | 95.2% | 0.73 (0.62-0.85) |
| Repetitive variable decelerations >60 s with baseline >160 or variability <5 between dec >20 min | 95.2% | 0.33 (0.19-0.46) |
| Prolonged deceleration ≥5 min (before the last 10 min) | 94.7% | 0.75 (0.69-0.81) |
| ≥3 prolonged decelerations 3–5 min | 95.6% | 0.45 (0.32-0.58) |
| ≥50% below baseline during 30 min due to decelerations (1) | 88.8% | 0.72 (0.68-0.77) |
| **Classification pathological** (any of the criteria above identified) | 87.3% | 0.74 (0.70-0.78) |
