# Supplementary material for: An attempt to improve recognition of fetal acidemia by a remodeled intrapartum cardiotocography classification: A case–control study
Source: Acta Obstet Gynecol Scand. 2026 Jun 10:10.1111/aogs.70287. Online ahead of print. doi: 10.1111/aogs.70287 (PMC13394735; doi:10.1111/aogs.70287)
Supplement: Supplementary file 1 — Table S1. Sensitivity and specificity of three current CTG interpretation guidelines to identify neonates with acidemia, after the investigational addition of the criterion “fetal heart rate >50% below the baseline during 30 minutes” to the respective guideline. For the proposed Model 1, this criterion is included as one of the features defining a “pathological” pattern. [file AOGS-9999-0-s001.docx]

|  | Cases  (N=364) | Cases with  50% below baseline | Controls  (N=728) | Controls with 50% below baseline | Sensitivity  with ”50% below baseline” added (%) | Specificity  with ”50% below baseline” added (%) |
| --- | --- | --- | --- | --- | --- | --- |
| TOTAL | 364 | 227 | 728 | 78 |  |  |
| **FIGO** |  |  |  |  | 73.9 (69.1–78.3) | 85.0 (82.2–87.5) |
| *Pathological* | 192 | 150 | 56 | 25 |  |  |
| *Suspicious* | 146 | 69 | 408 | 45 |  |  |
| *Normal* | 26 | 8 | 264 | 8 |  |  |
| **NICE** |  |  |  |  | 89.8 (86.3–92.7) | 69.1 (65.6–72.4) |
| *Pathological* | 318 | 218 | 214 | 67 |  |  |
| *Suspicious* | 32 | 7 | 241 | 10 |  |  |
| *Normal* | 14 | 2 | 273 | 1 |  |  |
| **SWE** |  |  |  |  | 76.1 (71.4–80.4) | 83.9 (81.1–86.5) |
| *Pathological* | 213 | 163 | 66 | 27 |  |  |
| *Suspicious* | 54 | 25 | 69 | 20 |  |  |
| *Normal* | 97 | 39 | 593 | 31 |  |  |
| **Model 1** |  |  |  |  |  |  |
| *Pathological* | 315 | 227 | 160 | 78 | 86.3 (82.3–89.6) | 77.9 (74.7–80.9) |

**Supplementary Table 1.** Sensitivity and specificity of three current CTG interpretation guidelines to identify neonates with acidemia, after the investigational addition of the criterion “fetal heart rate >50% below the baseline during 30 minutes” to the respective guideline. For the proposed Model 1, this criterion is included as one of the features defining a “pathological” pattern.
