# Supplementary material for: An attempt to improve recognition of fetal acidemia by a remodeled intrapartum cardiotocography classification: A case–control study
Source: Acta Obstet Gynecol Scand. 2026 Jun 10:10.1111/aogs.70287. Online ahead of print. doi: 10.1111/aogs.70287 (PMC13394735; doi:10.1111/aogs.70287)
Supplement: Supplementary file 2 — Table S2. Agreement between two assessors in interpretation of different CTG patterns included as “pathological” in Model 1, and agreement in classification pathological according to the new model. All 1092 traces are included except for the variable “Variability <5 bpm without accelerations ≥50 min” for which only registrations of 60 min were included. [file AOGS-9999-0-s002.docx]

| Table S2 Agreement between two assessors in interpretation of different CTG patterns included as ´pathological´ in Model 1, and agreement in classification pathological according to the new model. All 1092 traces are included except for the variable “Variability <5 bpm without accelerations ≥50 min” for which only registrations of 60 minutes were included. | | |
| --- | --- | --- |
|  | **Agreement  (%)** | **Kappa index**  **(95% CI)** |
| Baseline >170 bpm | 98.0% | 0.74 (0.63-0.84) |
| Baseline <100 bpm | 97.7% | 0.74 (0.64-0.84) |
| Variability <5 bpm without accelerations ≥50 min | 96.9% | 0.59 (0.45-0.73) |
| Variability <2 bpm ≥5 min | 97.2% | 0.76 (0.68-0.74) |
| Variability >25 bpm ≥10 min | 93.6% | 0.24 (0.13-0.35) |
| ≥5 late decelerations during 1 h | 90.9% | 0.66 (0.60-0.72) |
| ≥5 combined decelerations during 1 h | 92.6% | 0.53 (0.43-0.62) |
| ≥3 variable dec >60 s with variability <2 bpm within the decelerations (1) | 95.2% | 0.73 (0.62-0.85) |
| Repetitive variable decelerations >60 s with baseline >160 or variability <5 between dec >20 min | 95.2% | 0.33 (0.19-0.46) |
| Prolonged deceleration ≥5 min (before the last 10 min) | 94.7% | 0.75 (0.69-0.81) |
| ≥3 prolonged decelerations 3–5 min | 95.6% | 0.45 (0.32-0.58) |
| ≥50% below baseline during 30 min due to decelerations (1) | 88.8% | 0.72 (0.68-0.77) |
| **Classification pathological** (any of the criteria above identified) | 87.3% | 0.74 (0.70-0.78) |
